# Immersion to impact: does one or three years of rural immersion influence graduate clinical practice intentions and locations?

**DOI:** 10.3389/fmed.2025.1587912

**Published:** 2025-07-23

**Authors:** Will Harvey, Zahra Ali, Lachlan Van Schaik, Tamekha Develyn, Julian Wright

**Affiliations:** Department of Rural Health, The University of Melbourne, Shepparton, VIC, Australia

**Keywords:** rural medical workforce, medical education, rural immersion, workforce retention, rural clinical school

## Abstract

**Background:**

Addressing rural healthcare workforce shortages requires evidence-based strategies in medical education. Extended rural immersion programs offer a potential solution, but the optimal duration for fostering long-term regional and rural practice remains unclear.

**Methods:**

This retrospective study evaluates the impact of one-year versus three-year rural immersion experiences at the University of Melbourne’s Rural Clinical School (RCS) on graduate clinical practice locations of the 2016–2023 graduating cohorts. Using logistic regression analysis, we assessed key predictors of regional and rural practice, including rural immersion duration and intent to practice regionally or rurally.

**Results:**

Graduates who completed the three-year rural immersion program were significantly more likely to practice in Modified Monash Model (MMM) 2–7 areas than those with only one year of rural immersion. Intent to practice regionally or rurally and completing a regional/rural internship emerged as strong predictors of regional/rural practice. However, regional/rural intent did not appear to be a strong indicator for students who only completed 1-year of rural immersion. This highlights the importance of the duration of immersion.

**Conclusion:**

The study demonstrates the effectiveness of extended rural immersion in increasing regional and rural workforce retention. Findings support further investment in rural medical education, including end-to-end rural training models, which integrates rural exposure across the entire medical education journey. Future research should examine long-term workforce retention and strategies for sustaining rural career pathways.

## Introduction

Australia has many sparsely populated rural communities, with around 28% of the Australian population living in rural and remote areas (1). However, despite Australia’s highly trained workforce, equitable access to healthcare remains a critical issue in rural Australia (2).

The ongoing issue of health workforce maldistribution, particularly primary care practitioners, is the foremost challenge associated with health service delivery in many rural communities. This has a compounding effect on health outcomes for rural patients with increasing burden of disease based on remoteness and population size (3, 4). The Modified Monash Model (MMM) is used as a measure of remoteness and population size. These range from MMM1 to MMM7, where MMM1 is a major city and MMM7 is a very remote community (5). The distance and travel time have been implicated in adverse health outcomes and increasing mortality rates for rural communities (1, 6). In response, The Department of Health acknowledged the chronic shortage of rural health workforce and in its 2021–2023 Australian Medical Workforce Strategy report, one of the priorities identified was the rebalancing of supply and distribution of the workforce (2). In addition, a key policy recommendation by World Health Organization (WHO) adduced that student selection and education opportunities are some of the ways to address this global issue.

One concrete strategy being employed to influence the distribution of healthcare workers to rural areas is through rural clinical placements. Integrating these placements, as part of a nursing or allied health degrees, can positively affect students’ intent for future regional or rural practice (7). Additionally, rurally based clinical placements that are integrated into a medical degree have a positive impact on regional/rural practice intent (8), hence rural educational opportunities form key policy focuses for the World Health Organization (9), Australian government, and medical schools. Embedding students in rural communities, with full immersion in placements, goes one step further and is a strong predictor of rural practice (10). This is corroborated from student tracking data from nine Australian medical programs with rural clinical schools (RCS), with graduates who undertook extended RCS placements 2.93 and 1.76 times more likely to be a general practitioner (GP) or non-GP specialist, respectively, in a non-metropolitan area (MMM2-7) (4).

**FIGURE 1:**
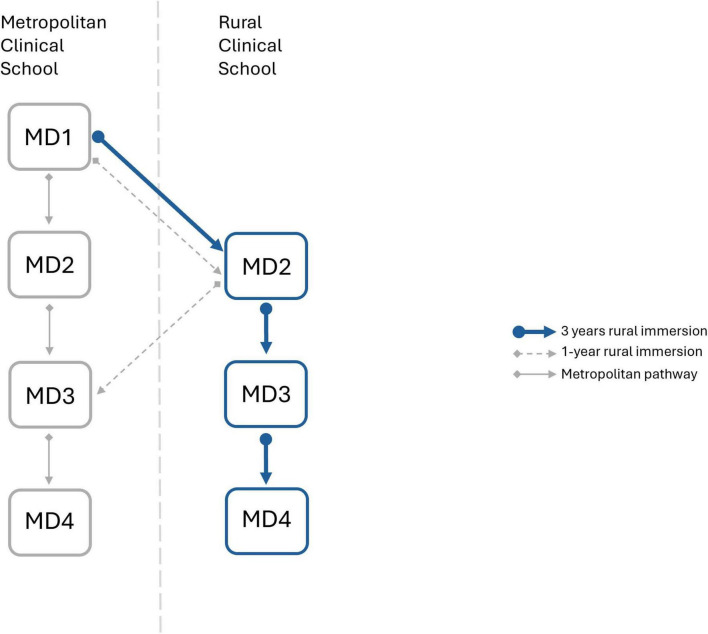
Schematic representation of the rural immersion programme at the University of Melbourne’s Rural Clinical School. Three years of rural immersion (MD2, MD3, and MD4) is represented with blue arrows, 1-year rural immersion (MD2 only) is represented by dashed gray arrows, metropolitan pathway is represented by solid gray arrows.

**FIGURE 2:**
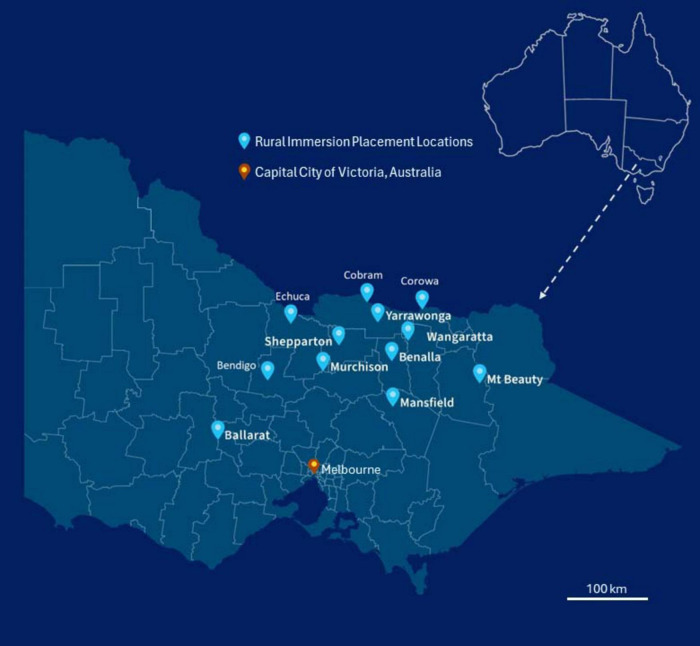
The University of Melbourne, Doctor of Medicine rural immersion program placement locations across rural Victoria (MMM2-MMM7). 2019 MMM Classifications of Rural Immersion Placement Locations; Ballarat (MMM2), Benalla (MMM4), Bendigo (MMM2), Cobram (MMM4), Corowa (MMM4), Echuca (MMM3), Mansfield (MMM5), Mt Beauty (MMM5), Murchison (MMM5), Shepparton (MMM3), Wangaratta (MMM3), Yarrawonga (MMM4) (5).

The extended rural placement of students (rural immersion) at the University of Melbourne’s Rural Clinical School (RCS) was an immersive program designed to provide clinical placements and community integration to students that had an interest in rural health (11). Existing University of Melbourne Doctor of Medicine (MD) Students submitted an expression of interest to join the rural cohort and were not required to meet any prerequisite criteria relating to rurality or academic performance. Students accepted into the cohort (Figure 1) could preference placement location from several primary healthcare settings across regional and rural Victoria (MMM2-MMM5) (Figure 2). The breadth of exposure allowed students to experience a diversity of clinical educational opportunities including skills in general practice, women’s health, child and adolescent health, aged care, and mental health. The program was designed with central theme of rural immersion, intended to further strengthen the long-term positive outcomes on medical workforce within these regional and rural communities. This paper aims to retrospectively assess duration of rural immersion (1 or 3 years), together with intent for future regional or rural practice, in order to determine their impact on influencing graduates between 2016–2023 to undertake regional or rural clinical practice.

## Methods

### Participants

The study design and analysis were approved by the University Human Ethic Committee (HEC30266). Administrative data was collected by survey (distributed via institutional email lists) between 2016 to 2023 to inform the RCS of graduates’ place of internship, their future intentions of location for employment (expressed as MMM) and their interests in regional/rural practice. Participant consent was implied through voluntary completion of the survey. The participants were graduating medical students.

### Data collection

Data collected were full name, MD4 locations and MMM classification, internship location along with preference number. In addition, graduating students were also asked of their intent, particularly about their future training interests, whether they were interested in a regional/rural career following the rural immersion programme and a potential 10-year plan.

Australian Health Practitioner Regulatory Agency (AHPRA) data were cited via the online site (12). Each graduate was individually searched, and the following data was recorded: AHPRA number, date of registration, profession, registration type and status, principal place of practice and any specialist registration and year fellowed.

Subsequently, MMM classifications were also assigned to graduates’ MD4 location, internship location and principal place of practice (as of October/November 2024), identified via the AHPRA website.

### Data analysis

Primary data analysis and generation of descriptive statistics was performed in Microsoft Excel [Microsoft 365 MSO (Version 2411 Build 16.0.18227.20082) 64-bit]. Logic checks were performed, and crosstabs generated to ensure data accuracy. Binomial logistic regression was performed in SPSS [Version 30.0.0.0 (172)] on raw data output from Excel. Using the data, the model was used to assess the effect of independent variables: association between enrolment in the rural immersion cohort, graduating year and intent, on whether graduates ultimately, practiced in a metro or regional/rural location.

Further, assessment of performance of the binomial logistic regression model was done using the ROC curve. Figures and the two-way ANOVA analysis was generated in GraphPad Prism [Prism 10 for Windows 64-bit Version 10.3.1(509)] to assess the effects of individual independent variables against dependent variable. The alpha was set at 0.05.

Descriptive statistics (percentages calculated for each cohort, mean calculated from percentages, variance represented by Standard Error of Mean; SEM) was used to illustrate the demographics of students surveyed. The responses elucidated diversity of students, distribution of gender during each graduating year and intent in regional or rural practice. The surveys also identified their MD4 location, however, for 2021 cohort, specific MD4 locations were not recorded (of internship towns). Hence, this independent variable was collapsed to inform whether the graduates completed 1-year or 3 years of the rural immersion programme (Figure 1). Graduates also listed their internship preference level on a numerical scale, “1” being their first preference and increasing numbers indicating decreasing levels of preference. Data extracted from APHRA were the registration type, principal place of practice and speciality attained.

For all locations, the Modified Monash Model was identified based on the 2019 classification (5).

### Variable overview for binomial logistic regression model

For anonymity, data was converted to numbers with levels nested in each variable. For “graduate year,” variable was collapsed into two levels: PGY ≥ 5 and PGY < 5. Response to “Intention to practice rurally” was adjusted to three levels; “Yes,” “No” and “Maybe/Unsure.” “Duration of rural placement” was collapsed into binary levels: 1-Year rural immersion or 3-Years rural immersion. MMM classification of MD4 location, internship placement location (represented as MMM) was also binarised to “Metro” (MMM1) and “Rural” (MMM2-7) levels of variables. While MMM2 locations are classified by the Department of Health and Aged Care as “regional” rather than “rural,” they are commonly included in rural medical workforce initiatives, and thus have been grouped with MMM classifications 3 to 7. Independent variables such as “Gender” had two levels Male and Female; “Registration type” had five levels (Provisional, General, General Specialist, Specialist, and non-practising), with “Speciality” also recorded from AHPRA. “Principal place of practice” was the dichotomised (Metro/Rural) dependent variable.

## Results

### Demographics

**FIGURE 3:**
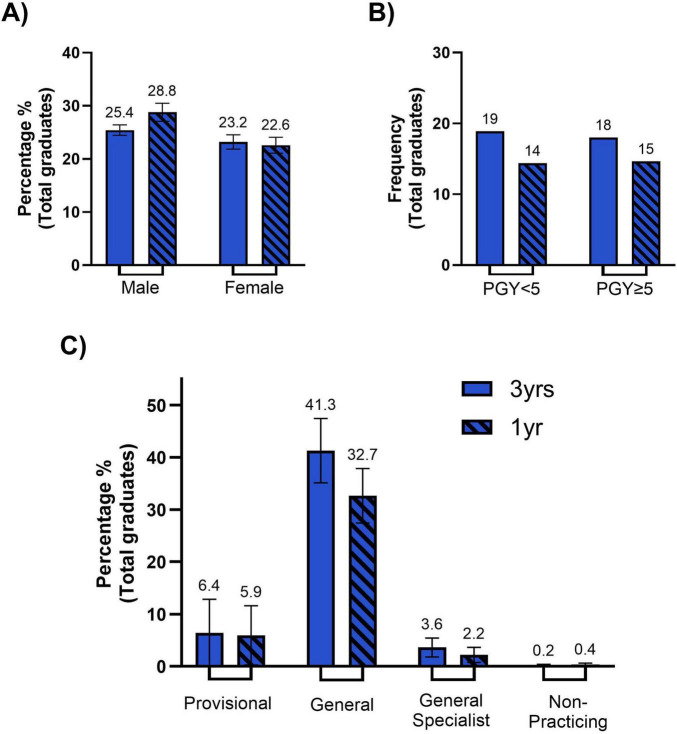
Demographics of our student cohort. **(A)** Bar chart representing the percentage of our student cohort separated by those who were in the 3-year immersion program (solid color) versus those who a 1-year immersion program (diagonal lines), that are male (dark blue) and female (light blue). The percentage of males (left) and females (right) amongst are statistically comparable across the 3-year and 1-year immersion programs. Error bars represent the standard error of the mean (SEM) **(B)** Bar chart representing the frequency of 3-year immersion (solid color) and 1-year immersion (diagonal lines) student cohort that are currently identified as being in a post graduate year (PGY) < 5 years (dark blue) or PGY ≥ 5 years (light blue). Whilst the total frequency of 1-year immersion graduates for PGY < 5 and PGY ≥ 5 were slightly lower than 3-year immersion graduates, they are statistically comparable. **(C)** Bar chart representing the percentage of total graduates that are currently registered as provisional (dark blue), general (medium blue), general specialist (light blue) or non-practicing (gray), divided by 3-year immersion (solid color) and 1-year immersion (diagonal lines) cohorts. No statistical significance is seen between 3-year and 1-year immersion cohorts for any registration type (provisional, general, general specialist, non-practicing). Although not depicted on the graph significance is seen between the percentage of total graduates registered as general and all other registration types (for both 3-year immersion and 1-year immersion cohorts). Error bars represent the standard error of the mean (SEM).

This retrospective study focused on exit survey data of eight cohorts of medical students, who graduated in 2016 to 2023, and had undertaken a minimum of 1-year rural placement during their degree. This yielded a total of 537 graduates across the eight cohorts. At the time of this study, four of these eight cohorts were postgraduate year (PGY) 5 or greater (*n* = 266), and the remaining four cohorts were < PGY5 (*n* = 271) (Figure 3B). Given the limited time since graduation, only a small proportion of graduates had completed specialty training (5.8%). The majority of graduates possessed a general (73.6%) or provisional (12.8%) registration (Figure 3C). Of the total graduates 47.9% were male and 52.1% were female (Figure 3A), 54.9% completed three years of rural immersion (3 years rural immersion) and 45% completed one-year of rural immersion. AHPRA records could not be located, and hence a principal place of practice could not be determined, for 42 of the 537 graduates.

### Binomial logistic regression outcome

The logistic regression model was statistically significant [χ^2^(24) = 138.472, *p* < 0.001], indicating that the predictor variables as a group reliably distinguished between medical graduates practicing in regional/rural versus metropolitan areas. The model explained 30.8% of the variance in practice location based on the Cox & Snell *R*^2^ and 45% based on the Nagelkerke *R*^2^. The overall classification accuracy was 84.8%, with a sensitivity of 62.6% (correctly identifying regional/rural practitioners) and a specificity of 92.8% (correctly identifying metropolitan practitioners).

The performance of the logistic regression model was also evaluated using a receiver operating characteristic (ROC) curve (Supplementary Figures 1–3, Supplementary Tables 1–4). The area under the curve (AUC) was 0.857 (Supplementary Table 2), indicating very good discrimination between regional/rural and metropolitan practice locations. The ROC curve analysis supports the utility of the logistic regression model in identifying significant predictors of regional/rural practice location, with sensitivity and specificity aligning with the overall classification accuracy presented earlier (84.8%).

Among the predictor variables, internship location [B = 2.174, Exp(B) = 8.793, *p* < 0.001] and intention to practice regionally/rurally [B = 1.789, Exp(B) = 5.984, *p* = 0.026] emerged as significant contributors (Table 1). Graduates who completed their internship in a regional/rural location were nearly nine times more likely to currently practice in a regional or rural area compared to those who interned in metropolitan areas. Similarly, graduates who had expressed an intention to practice regionally/rurally during their studies were approximately six times more likely to be practicing regionally or rurally.

**TABLE 1:** Results of binomial logistic regression analysis for predictors of rural practice location.

Variable	Odds ratio (95% CI)	*p*-value (alpha set at 0.05)
Placement duration (3 years, rural immersion)	1.03 (0.40–2.66)	0.947
Internship location (regional/rural)	8.79 (4.47–17.29)	< 0.0001*
**Regional/rural intent (overall effect)**	–	0.01*
Regional/rural intent (yes)	5.98 (1.24–29.01)	0.026*
Regional/rural intent (unsure)	2.25 (0.47–10.85)	0.314
Registration type (overall effect)	–	0.016*
Registration type (provisional)	0.28 (0.01–7.26)	0.279
Registration type (general)	0.17 (0.01–3.92)	0.172
Registration type (general specialist)	1.09 (0.04–28.43)	0.961
PGY (≥ 5 years)	0.52 (0.25–1.08)	0.081
Gender (female)	1.35 (0.73–2.50)	0.347
MD4 location (overall effect)	–	0.73
MD4 location (1)	0.94 (0.35–2.52)	0.91
MD4 location (2)	0.98 (0.32–2.99)	0.974
MD4 location (3)	0.84 (0.26–2.81)	0.778
MD4 location (4)	2.02 (0.63–6.46)	0.225
Internship preference (overall effect)	–	0.914
Internship preference (1)	1.02 (0.42–2.48)	0.96
Internship preference (2)	0.23 (0.06–0.89)	0.034*
Internship preference (3)	0.69 (0.16–2.97)	0.616
Internship preference (4)	0.76 (0.15–3.73)	0.734
Internship preference (5)	0.61 (0.13–2.93)	0.536
Internship preference (6)	0.00 (0.00–0.00)	1
Internship preference (7)	0.49 (0.04–5.99)	0.574
Internship preference (8)	1.00 (0.07–13.42)	0.997
Internship preference (9)	0.00 (0.00–0.00)	0.999
Internship preference (10)	0.00 (0.00–0.00)	1
Internship preference (11)	0.00 (0.00–0.00)	1
Constant	0.19	0.358

*p < 0.05. Bold font is to help emphasise statistically significant *p*-values.

Registration type was significant overall (*p* = 0.016), however, no individual registration type was found to be a useful predictor of practice location [Provisional (1): B = −1.277, Exp(B) = 0.279, *p* = 0.443] [General (2): B = −1.758, Exp(B) = 0.172, *p* = 0.270] [General Specialist (3): B = 0.082, Exp(B) = 1.086, *p* = 0.961]. Other variables, including gender (*p* = 0.347), placement duration (*p* = 0.947), and MD4 placement location (*p* = 0.730), did not significantly predict regional/rural practice. Internship preference levels showed variability, but extreme coefficients in some categories (e.g., preference levels 6, 9, 10 and 11 B = ∼20) reflect data sparsity. The odd ratios and 95% confidence intervals (CI) for all variables included in the logistic regression are presented in Table 1.

### ANOVA, Mann–Whitney test and simple linear regression

#### Two-way ANOVA

In conjunction with the logistic regression, a two-way ANOVA was conducted to examine the effects of the duration of rural placement (1-year vs. 3-years) and intention for regional/rural practice (yes vs. no vs. maybe/unsure) on the principal place of practice (metro vs. rural) of graduates. Tukey’s *post-hoc* test was used for pairwise comparisons. In a univariate model the main effect of placement duration (3-years vs. 1-year) was not statistically significant, F (1, 42) = 3.828, *p* = 0.057, indicating that graduates from the 3 years rural immersion were not more likely to choose regional or rural practice compared to those who completed only 1 year. The main effect of intention for regional/rural practice was significant (in a univariate model), *F*(2, 42) = 23.15, *p* < 0.0001, with graduates who expressed positive intent for regional/rural practice more likely to practice in regional/rural areas compared to those who were unsure or had no intention to practice regionally/rurally. The interaction between duration and intent was also significant, *F*(2, 42) = 16.06, *p* < 0.0001, suggesting that the effects of the 3 years rural immersion program and intention for regional/rural practice were dependent on each other.

#### Tukey’s multiple comparisons test

**FIGURE 4:**
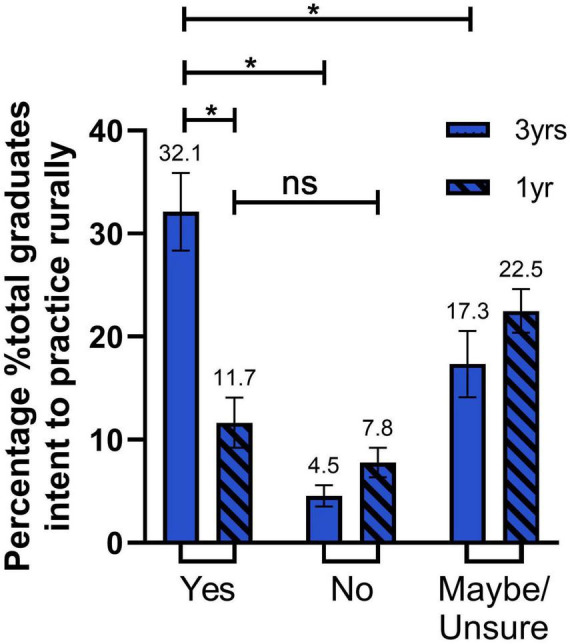
Graduates intent to practice regionally/rurally. Bar graph representing the percentage of total graduates’ intention to practice regionally/rurally; Yes (dark blue), No (medium blue), Maybe/Unsure (light blue) for 3-year rural immersion (solid color) and 1-year rural immersion (diagonal lines) cohorts. The percentage of 3-year immersion students with Yes intent compared to 1-year immersion students is statistically higher (*P* < 0.0001). There is a significantly lower percentage of 3-year immersion students who have a No or Maybe/Unsure intent compared to Yes intent (*P* < 0.0001, *P* = 0.0021, respectively). No significance is seen between 1-year immersion students with Yes or no intent (*P* = 0.8834). Error bars represent the standard error of the mean (SEM). Statistical significance was determined by two-way ANOVA multiple comparisons. *P ≤ 0.05.

Following the significant main effect for intent and interaction effect, Tukey’s multiple comparisons test revealed the following differences:

- Graduates of the 3 years rural immersion who expressed positive intent for regional/rural practice were significantly more likely to choose regional or rural practice compared to the 1-year rural immersion graduates who had also indicated positive intent (*p* < 0.0001) (Figure 4).

- Graduates of the 3 years rural immersion who expressed positive intent for regional/rural practice were significantly more likely to choose regional or rural practice compared to graduates of the 3 years immersion who indicated they were unsure (*p* = 0.0021) or that did not intend to practice regional/rurally (*p* < 0.0001) (Figure 4).

- 1-year rural immersion graduates who expressed positive intent for regional/rural practice were not more likely to practice regionally or rurally compared to 1-year rural immersion graduates that indicated no intention for regional/rural practice (*p* = 0.8834) (Figure 4).

#### Mann–Whitney U test

**FIGURE 5:**
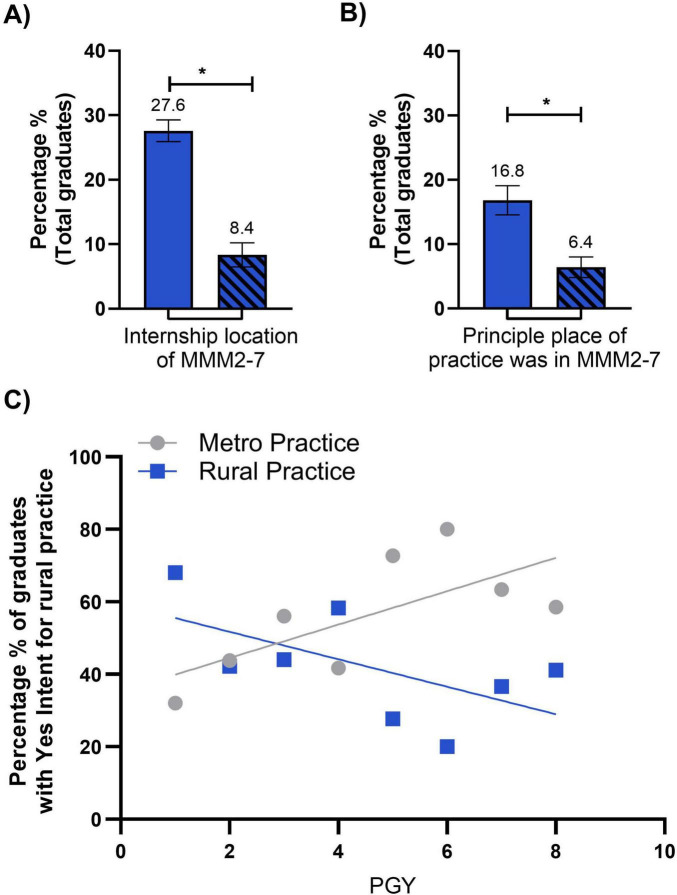
**(A)** Bar graph representing the percentage of total graduates in the 3-year rural immersion (solid color) and 1-year rural immersion (diagonal lines) cohorts whose internship location was zoned within MMM2-7. The percentage of 3-year immersion students with an internship location in MMM2-7 was significantly higher than 1-year immersion students (*P* = 0.0006). Error bars represent standard error of the mean (SEM). **(B)** Bar graph representing the percentage of total graduates in the 3-year immersion (solid color) and 1-year immersion (diagonal lines) cohorts whose principal place of practice was zoned within MMM2-7. The percentage of 3-year immersion students whose principal place of practice was located within MMM2-7 was significantly higher than 1-year immersion students (*P* = 0.0019). Error bars represent standard error of the mean (SEM). Statistical significance for **(A,B)** was determined by Mann-Whitney test. *P < 0.05. **(C)** Liner regression highlighting those students who are metro (gray circles) and rural (blue squares) practicing who had a Yes intent for regional/rural practice, across the 8 post graduate years (PGY). Metro practicing students across the PGY’s shows a positive trend and Rural practicing shows a negative trend across PGY’s, statistical analysis revealed no significant difference between the slopes of the two regression lines (*P* = 0.0559, *P* = 0.1141, respectively). Statistical significance was determined by Mann-Whitney test of percentage. *P < 0.05.

A Mann–Whitney U test was conducted to compare the regional/rural internship location between graduates of the 3 years rural immersion program and those who completed only a 1-year rural immersion placement. Results showed a significant difference in rural internship locations between the two groups (*U* = 0, *p* = 0.0006, two-tailed) (Figure 5A). Graduates with 3 years rural immersion had a higher sum of ranks (77) compared to graduates who completed a 1-year rural placement (sum of ranks = 28). The median regional/rural internship location for 3 years rural immersion graduates was 29.58 (*n* = 7), significantly greater than the median of 7.14 for 1-year rural immersion graduates (*n* = 7). The actual difference in medians was −22.44, with the Hodges–Lehmann estimate of the difference being −19.24, further highlighting the disparity in regional/rural internship locations between the two groups.

A Mann–Whitney U test was conducted to compare principal place of practice (metropolitan or regional/rural) between graduates with 3 years rural immersion and those who completed only 1-year of rural immersion. Results showed a significant difference in practice locations between the two groups (*U* = 4, *p* = 0.0019, two-tailed) (Figure 5B). Graduates with 3 years of rural immersion had a higher sum of ranks (96) compared to graduates who completed a 1-year rural immersion (sum of ranks = 40). The median regional/rural internship location for 3 years rural immersion graduates was 17.47 (*n* = 8), significantly greater than the median of 5.66 for 1-year rural immersion graduates (*n* = 8). The actual difference in medians was −10.81, with the Hodges–Lehmann estimate of the difference being −10.57. These results highlight the disparity in principal place of practice between the two groups, albeit this is not as pronounced as internship location.

#### Simple linear regression

Graduates with intention for regional/rural practice (regardless of placement duration) were grouped and their principal place of practice was presented as a function of time since graduation (Figure 5C). These data demonstrate a potential trend of rural attrition (Slope = −3.772, *R*^2^ = 0.3566) and metropolitan accumulation (slope = 4.609, *R*^2^ = 0.4826). However, neither slope was statistically significant (*p* = 0.118 and *p* = 0.056, respectively).

## Discussion

The findings from the extended rural placement of students (rural immersion) at the University of Melbourne’s Rural Clinical School (RCS) provide evidence of the program’s success in addressing regional and rural healthcare workforce shortages. By implementing rural immersion and emphasizing regional and rural placements, the 3-year rural immersion program has achieved significant outcomes. Graduates who were part of the full 3-year immersion were significantly more likely to intend on practicing in a regional or rural location, than those who only completed 1-year (Figure 4). Additionally, graduates who indicated intention for regional/rural practice were almost 6 times more likely to be currently practicing in a regional or rural location (Table 1). These results highlight the program’s effectiveness in encouraging medical graduates to serve in rural and remote areas, thereby contributing to goal of equitable healthcare distribution. The results highlight that intent to practice regionally/rurally, and regional or rural internship (MMM2-MMM7) are significant predictors of eventual regional or rural practice (Figures 4, 5A, B). This aligns with previous studies that have demonstrated the correlation between rural exposure during clinical training and rural workforce retention (4, 13). Interestingly, there is evidence to support strategies to enhance intention to practice rurally in nursing and allied health students that are not simply restricted to rural origin students in order to build rural workforce. The results of this study support findings from other studies assessing whether rural clinical placements influence a change in intention to practice regionally or rurally. Glenister et al. (7) report that rural origin and rural training are significant predictors of working regionally or rurally, and that metropolitan based students can change their intention to practice rurally after a rural placement. This is in-line with our results that intent to practice regionally/rurally and internship placement in regional/rural settings are significant predictors of eventual regional or rural practice. We do not have data on what proportion of graduates are of rural origin, however, our new end-to-end training program admits prospective students based on rural origin, among other variables.

As seen in Figure 5C, our data demonstrate a potential rural attrition rate and metropolitan accumulation. It could be argued that there is a loss of rural graduates to metropolitan areas for specialist vocational college-led training. General practice (GP) specialty has largely had a good uptake from our 3 years rural immersion graduates (Figure 3C). In a study of associations between specialty type and practice location at PGY10 from a cohort of nine Australian universities (4), at PGY10, two thirds (820/1220) had achieved fellowship. Furthermore, GP’s were 2.8 times more likely to be in non-metropolitan practice than graduates with all other specialist qualification (4). In terms of rural medical workforce, there continues to be limited numbers of general practitioners and medical specialists in rural and regional Australia. The growing trend toward sub-specializations is resulting in a shortage of generalists, particularly in regional and rural Australia (14, 15). There is a need for funded rural and regional internships, rural generalist and specialist training posts and pathways. Government initiatives are well-placed to increase the delivery of vertically integrated medical education. Examples include the Murray to the Mountains intern program, the Mt Gambier community-based junior doctor program, and the Monash Gippsland health education model. To address the shortage of training opportunities in rural and regional Victoria, the University of Melbourne, Deakin University, and Monash University have worked to develop these cost-effective and sustainable solutions. However, in addition to this, there is a need for the medical colleges in Australia to continue increasing the number of specialist vocational training opportunities to be undertake regionally and therefore increase rural training opportunities. As there is a clear relationship between the importance of regional and rural training pathways to longer term work outcomes, and a need to expand specialist vocational training that supports more rural training opportunities. This will lead to the much-needed increase in regional and rural medical workforce after the internship years.

The statistical methodology employed in this study demonstrates its strength and reliability. By utilizing a logistic regression model that accounts for multiple variables simultaneously, the analysis effectively captures the intricate and multifaceted factors influencing regional/rural practice intentions. This approach is critical for mitigating the effects of confounding variables, ensuring that the associations identified are robust and meaningful. Unlike methods such as ANOVA or the Mann–Whitney test, logistic regression also allows for the assessment of model performance, including sensitivity and specificity, providing a more comprehensive evaluation of predictive accuracy.

However, the lower predictive performance of our model in determining regional or rural practice highlights a persistent challenge for institutions: selecting and admitting the most suitable candidates into rural medical programs. While individual institutions may strive to identify students with strong rural intent, national workforce outcomes are unlikely to improve without broader collaboration. Rural medical programs must therefore prioritize data sharing to enhance the translation of intent into actual regional or rural practice. At the same time, it remains essential to preserve other key selection priorities, such as diversity in student backgrounds and experiences, as personality plays a part in an individual’s future practice location (16). This highlights the need for continued refinement of recruitment strategies to ensure rural programs attract and support students most likely to commit to, and thrive in, regional and rural practice.

This rigorous methodology enables nuanced insights into key predictors, such as regional or rural internship placements and intent to practice regionally or rurally, both of which are strongly linked to long-term regional/rural workforce retention (Figures 4, 5A, B). These attributes provide a solid foundation for evidence-based decision-making and highlight the importance of employing advanced statistical techniques when evaluating the impact of programs like the extended rural immersion. Unfortunately, this level of methodological rigor has not always been achieved in previous studies assessing rural placements.

The University of Melbourne’s new end-to-end medical program, funded through the Australian Government’s Murray-Darling Medical Schools Network (MDMSN), builds upon the rural immersion program’s foundation. This initiative offers several advancements such as, comprehensive training, enhanced support, and potentially improve outcomes in terms of workforce retention, as evidenced by the higher proportion of 3 years rural immersion students with regional/rural intent then converting to rural practice (Figures 4, 5A, B). Additionally, the novel approach to medical education is it being entirely within regional/rural settings, therefore program eliminating any transitional phases that could disrupt regional/rural practice intentions. Additionally, the now MDMSN-funded program aligns with the latest national priorities to address regional and rural workforce shortages in Australia, and in doing so ensures sustainable support for its objectives. It is too early to know the end-to-end model may further improve retention rates in regional and rural practice compared to the rural immersion program, however it undoubtedly integrates rural exposure throughout the entire medical education journey, and data collection evaluating the impact of the MDMSN is currently underway (17). It is clear that retention of medical graduates is key in increasing a sustainable rural medical workforce (13), thus increasing opportunities for extended rural placement is most likely critical in achieving this aim. The 3 years rural immersion program demonstrated an interaction effect, as evidenced in Figure 4 on 3 years participation and regional/rural intent. This suggests two key dynamics, either the program was strengthening students’ intent to practice regionally or rurally, or students with stronger pre-existing regional/rural intent were more likely to self-select into the 3 years programme. Building on these findings, the new end-to-end program strategically recruits students with strong pre-existing regional/rural intent. It then leverages the program structure to nurture, reinforce, and strengthen this intent while simultaneously fostering connections with regional and rural practices. This approach aims to facilitate a seamless transition into regional and rural internships, further solidifying the pathway to a sustained rural medical workforce. As such, the introduction of end-to-end rural medical training is a major step forward in terms of workforce retention.

The findings of this study suggest that sustained and comprehensive rural exposure, as provided by the rural immersion model, may support the development of continuity in students’ training experiences and professional relationships. This exposure appears to positively influence students’ intent to practise in regional or rural settings. The inclusion of a range of clinical disciplines within the rural curriculum is intended to prepare students for the diverse demands of rural healthcare delivery, although this was not directly assessed in this study. Additionally, the program’s approach of allowing students to express placement preferences may contribute to higher levels of engagement and commitment to rural training pathways.

A limitation of this study is the reliance on data from AHPRA and administrative sources presents potential inaccuracies in identifying graduates’ principal places of practice. This is due to AHPRA data potentially not reflecting the most current practice locations due to delays in updating principal practice information. This could misrepresent the graduates’ actual regional/rural or metropolitan practice distribution at any given time. Additionally, this could lead to missing data. Although this has not had a significant impact on this study, due to small numbers of missing data, but it could potentially impact previous studies with much larger cohorts. However, in Australia, AHPRA remains the best option logistically to do this. A closer working relationship with AHPRA may be required for future studies to ensure the best possible quality data.

Another important limitation relates to the use of the Modified Monash Model (MMM) in a binary form. In this study, MMM categories 2 to 7 were combined into a broader “regional/rural” grouping, which risks masking important distinctions between regional centers (MMM2), rural communities (MMM3–6), and remote areas (MMM7). These locations vary markedly in healthcare access, education access, employment opportunities for partners, infrastructure, and workforce retention challenges. Future research with larger sample sizes, or through collaborative data sharing across institutions, should aim to examine these categories separately to provide more nuanced insights.

Furthermore, the self-reported nature of intent data introduces an element of subjectivity and may not fully capture the multifactorial and evolving nature of decisions about future practice location. Mixed-methods approaches that incorporate both quantitative data and qualitative insights may better reflect the complexity of these decisions. Finally, the retrospective design of the study limits the ability to infer causality between training experiences and subsequent practice location.

The transition from the rural immersion program to the MDMSN-funded end-to-end medical program represents a strategic evolution in rural medical education. By addressing systemic barriers such as professional isolation and limited career development opportunities, the new program aims to create a resilient and regionally distributed healthcare workforce. Future research should evaluate the long-term impacts of this model, and longitudinal integrated clerkship (LIC) models more broadly. In particular, future work should examine their effectiveness in retaining graduates in regional and rural practice and its scalability to other institutions. Additionally, exploring the interplay of factors such as mentorship quality and community integration could provide deeper insights into shaping practice intentions. As addressing systemic barriers to rural practice, such as professional isolation, spousal opportunities, schooling options for children etc., remains essential.

## Conclusion

These findings demonstrate the evidenced based approach that can be taken to evolving and optimizing rural medical education programs and their impact on meeting the healthcare needs of regional and rural communities. A critical aspect of our study’s robustness lies in the statistical methodology employed. Utilizing a logistic regression model that controlled for multiple variables simultaneously, significant associations emerged regarding practice intent. This methodological distinction is crucial as it captures the complexity of factors influencing regional and rural practice decisions and underscores the importance of using advanced statistical methods to control for confounding variables. Future studies should adopt similar approaches to ensure accurate and meaningful interpretations of program outcomes.

Outcomes from the rural immersion program demonstrates its value in influencing medical graduates to practice in regional or rural areas. By integrating comprehensive rural immersion into medical education, the program addresses critical workforce shortages and contributes to the equitable health outcomes for those living in rural communities. These findings support continued investment in, and expansion of, rural clinical programs. Encapsulated by the new, evidenced-based end-to-end program, is designed to improve retention rates in regional and rural practice compared to the rural immersion program, as it recruits for rural origin students, with a strong intent for regional/rural practice, and now integrates regional and rural exposure throughout the entire medical education journey.

## Data Availability

The datasets presented in this article are not readily available because although raw data have been anonymized, group sizes are such that there may be a risk of reidentification of participants. As such, the dataset will not be made available. Additional ethical approval must be sought for use of this dataset, outside of the analyses presented here. Requests to access the datasets should be directed to w.harvey@unimelb.edu.au; lachlan.vanschaik@unimelb.edu.au.
