# Supplementary material for: Immersion to impact: does one or three years of rural immersion influence graduate clinical practice intentions and locations?
Source: Front Med (Lausanne). 2025 Jul 23;12:1587912. doi: 10.3389/fmed.2025.1587912 (PMC12325271; doi:10.3389/fmed.2025.1587912)
Supplement: Supplementary file 1 [file Data_Sheet_1.pdf]

Warnings

The test result variable(s): Predicted probability has at least one tie between the positive actual state group and the negative actual state group. Statistics may be biased.

Supplementary Table 1: Case Processing Summary

Binomial Outcome - Principal of practice	Valid N (listwise)
Positive[a]	99
Negative	278
Missing	160
Total	537

Larger values of the test result variable(s) indicate stronger evidence for a positive actual state.

a. The positive actual state is Rural.

Supplementary Figure 1: ROC Curve of Binomial Logistic Regression.

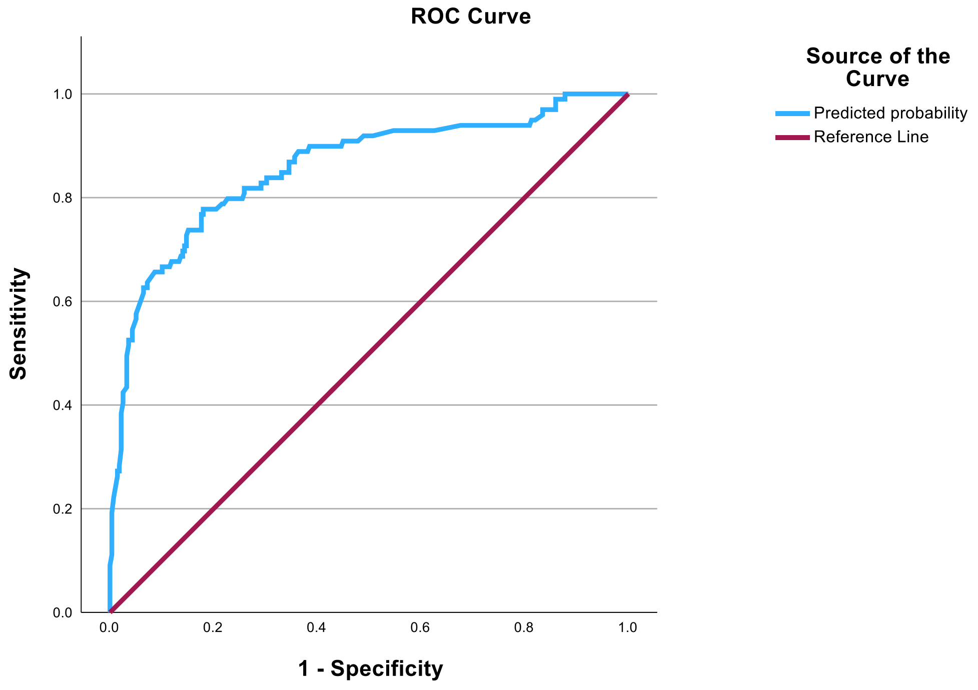

Supplementary Figure 2: Precision-Recall Curve of Binomial Logistic Regression.

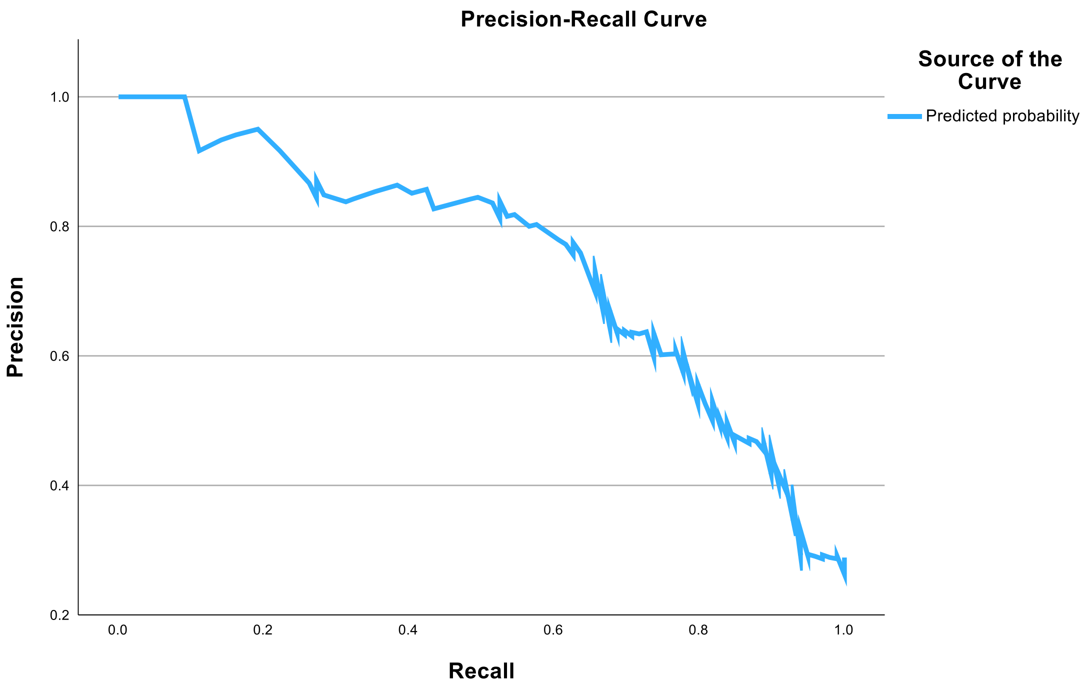

Supplementary Table 2: Area Under the ROC Curve

Test Result Variable(s): Predicted probability

Area	Std. Error[a]	Asymptotic Sig.[b]	Asymptotic 95% Confidence Interval	
			Lower Bound	Upper Bound
.857	.024	.000	.810	.904

The test result variable(s): Predicted probability has at least one tie between the positive actual state group and the negative actual state group. Statistics may be biased.

a. Under the nonparametric assumption

b. Null hypothesis: true area = 0.5

Supplementary Table 3: Classifier Evaluation Metrics

Test Result Variable(s): Predicted probability

Gini Index	K-S Statistics	
	Max K-S[a]	Cutoff[b]
.714	.598	.2792171

a. The maximum Kolmogorov-

Smirnov (K-S) metric. Also the maximum value of Youden's index.

b. In case of multiple cutoff values associated with Max K-S, the largest one is reported.

Supplementary Figure 3: Overall Model Quality of Binomial Logistic Regression

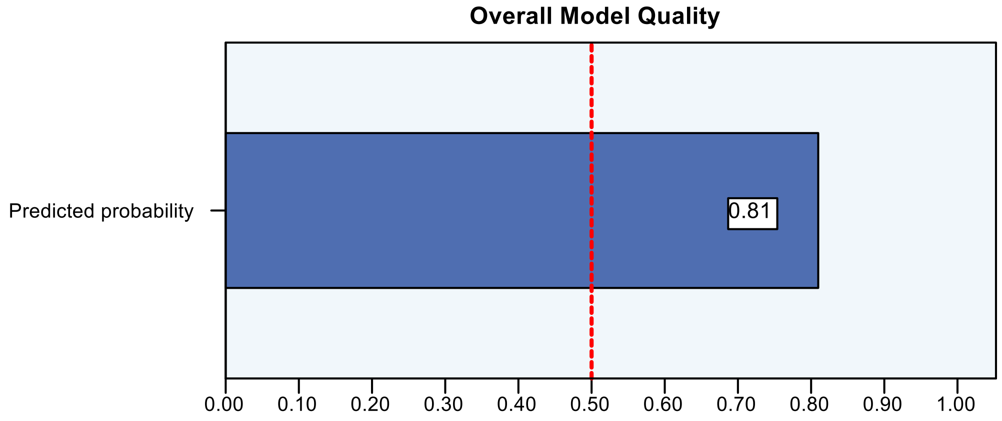

A good model has a value above 0.5
A value less than 0.5 indicates the model is no better than random prediction

Note: Use caution in interpreting this chart since it only reflects a general measure of overall model quality. The model quality can be considered "good" even if the correct prediction rate for positive responses does not meet the specified minimum probability. Use the classification table to examine correct prediction rates.

Supplementary Table 4: Coordinates of the ROC Curve

Test Result Variable(s): Predicted probability

Positive if Greater Than or Equal To[a]	Sensitivity	1 - Specificity	Youden's Index
.0000000	1.000	1.000	.000

.0000000	1.000	.996	.004
.0000000	1.000	.993	.007
.0000000	1.000	.989	.011
.0000000	1.000	.986	.014
.0000000	1.000	.982	.018
.0027009	1.000	.978	.022
.0064748	1.000	.975	.025
.0080815	1.000	.968	.032
.0087426	1.000	.964	.036
.0095008	1.000	.960	.040
.0108456	1.000	.957	.043
.0139226	1.000	.953	.047
.0163001	1.000	.946	.054
.0165165	1.000	.942	.058
.0167560	1.000	.939	.061
.0169449	1.000	.932	.068
.0174569	1.000	.928	.072
.0188865	1.000	.924	.076
.0211625	1.000	.921	.079
.0224200	1.000	.917	.083
.0224839	1.000	.914	.086
.0227598	1.000	.910	.090
.0231569	1.000	.892	.108
.0233001	1.000	.888	.112
.0234240	1.000	.885	.115
.0242466	1.000	.881	.119
.0252887	1.000	.878	.122
.0259961	.990	.878	.112
.0272686	.990	.871	.119
.0287561	.990	.867	.123
.0302193	.990	.863	.127
.0311665	.990	.860	.130
.0313342	.980	.860	.120
.0317166	.970	.860	.110
.0321708	.970	.856	.114
.0326980	.970	.849	.121
.0344528	.970	.845	.124
.0367575	.970	.835	.135
.0377524	.960	.835	.125
.0380695	.949	.820	.129

.0384611	.949	.813	.137
.0386476	.939	.809	.130
.0388046	.939	.802	.137
.0403892	.939	.799	.141
.0424414	.939	.791	.148
.0464924	.939	.770	.170
.0501046	.939	.766	.173
.0505762	.939	.745	.195
.0510856	.939	.741	.198
.0525917	.939	.737	.202
.0541273	.939	.730	.209
.0569866	.939	.727	.213
.0606658	.939	.723	.216
.0621504	.939	.716	.224
.0637980	.939	.712	.227
.0652454	.939	.709	.231
.0666482	.939	.705	.234
.0678707	.939	.691	.249
.0685964	.939	.680	.260
.0695400	.939	.676	.263
.0701493	.929	.626	.303
.0705625	.929	.622	.307
.0708158	.929	.615	.314
.0711006	.929	.612	.318
.0712592	.929	.604	.325
.0715180	.929	.583	.347
.0727043	.929	.579	.350
.0746537	.929	.576	.354
.0763916	.929	.572	.357
.0786027	.929	.565	.365
.0840938	.929	.561	.368
.0887284	.929	.558	.372
.0903351	.929	.550	.379
.0915465	.929	.547	.383
.0920471	.919	.507	.412
.0925771	.919	.504	.416
.0932282	.919	.489	.430
.0939516	.909	.478	.431
.0945489	.909	.475	.434
.0953072	.909	.464	.445

.0958533	.909	.460	.449
.0960744	.909	.457	.452
.0964505	.909	.453	.456
.0967559	.909	.450	.459
.0971251	.899	.446	.453
.0976813	.899	.442	.457
.0985581	.899	.439	.460
.1002101	.899	.432	.467
.1033424	.899	.424	.475
.1057083	.899	.421	.478
.1067903	.899	.417	.482
.1079359	.899	.414	.485
.1093653	.899	.385	.514
.1154563	.889	.381	.508
.1207488	.889	.367	.522
.1223251	.889	.363	.526
.1242811	.879	.356	.523
.1255230	.869	.356	.513
.1264622	.869	.353	.516
.1280489	.869	.349	.520
.1293187	.869	.345	.523
.1299856	.848	.345	.503
.1305890	.848	.342	.507
.1313108	.848	.338	.510
.1322591	.848	.335	.514
.1347056	.848	.331	.518
.1386775	.838	.331	.507
.1422953	.838	.309	.529
.1533854	.838	.306	.533
.1634257	.838	.302	.536
.1649856	.828	.302	.526
.1661987	.828	.299	.530
.1674890	.828	.295	.533
.1685694	.828	.291	.537
.1692324	.818	.291	.527
.1697429	.818	.288	.530
.1703379	.818	.281	.538
.1720882	.818	.273	.545
.1734984	.818	.266	.552
.1738642	.818	.263	.556

.1746211	.818	.259	.559
.1770759	.808	.259	.549
.1842968	.798	.255	.543
.1940705	.798	.252	.546
.2019293	.798	.245	.553
.2062599	.798	.241	.557
.2073854	.798	.234	.564
.2098232	.798	.230	.568
.2131314	.798	.227	.571
.2146376	.788	.219	.568
.2154883	.788	.216	.572
.2205655	.778	.205	.573
.2260370	.778	.201	.576
.2329311	.778	.198	.580
.2444875	.778	.194	.584
.2539777	.778	.191	.587
.2595217	.778	.187	.591
.2611766	.778	.183	.594
.2792171	.778	.180	.598
.3002100	.768	.180	.588
.3035239	.768	.176	.591
.3081280	.747	.176	.571
.3146022	.737	.176	.561
.3246728	.737	.173	.565
.3512872	.737	.169	.568
.3747312	.737	.165	.572
.3810276	.737	.162	.576
.3853675	.737	.158	.579
.3888299	.737	.155	.583
.3900009	.737	.151	.586
.3940855	.727	.147	.580
.3991164	.717	.147	.570
.4018337	.707	.147	.560
.4038109	.707	.144	.563
.4046521	.697	.144	.553
.4081460	.697	.140	.557
.4225216	.687	.140	.547
.4355768	.687	.137	.550
.4503735	.677	.133	.544
.4630843	.677	.129	.547

.4667465	.677	.122	.554
.4713060	.677	.119	.558
.4750237	.667	.115	.552
.4778586	.667	.112	.555
.4787203	.667	.101	.566
.4805479	.657	.101	.556
.4833864	.657	.097	.559
.4861051	.657	.086	.570
.4894495	.636	.072	.564
.5191096	.626	.072	.554
.5470189	.626	.068	.558
.5498335	.626	.065	.562
.5527543	.616	.065	.551
.5551442	.606	.061	.545
.5579501	.576	.050	.525
.5600540	.566	.050	.515
.5716879	.545	.043	.502
.5833005	.535	.043	.492
.5909026	.525	.043	.482
.6103948	.525	.040	.486
.6268979	.525	.036	.489
.6340852	.515	.036	.479
.6401179	.495	.032	.463
.6435382	.485	.032	.452
.6507608	.434	.032	.402
.6622657	.424	.025	.399
.6786414	.414	.025	.389
.6933633	.404	.025	.379
.6998839	.384	.022	.362
.7042923	.354	.022	.332
.7066550	.323	.022	.302
.7084215	.313	.022	.292
.7111131	.283	.018	.265
.7168714	.273	.018	.255
.7274227	.273	.014	.258
.7398770	.263	.014	.248
.7577018	.222	.007	.215
.7775918	.192	.004	.188
.7866765	.162	.004	.158
.7928612	.141	.004	.138

.8261636	.111	.004	.108
.8550280	.091	.000	.091
.8560820	.071	.000	.071
.8722453	.061	.000	.061
.8886180	.051	.000	.051
.9064908	.030	.000	.030
.9327749	.010	.000	.010
1.0000000	.000	.000	.000

The test result variable(s): Predicted probability has at least one tie between the positive actual state group and the negative actual state group.

a. The smallest cutoff value is the minimum observed test value minus 1, and the largest cutoff value is the maximum observed test value plus 1. All the other cutoff values are the averages of two consecutive ordered observed test values.
